# Clinical and Clinico-Pathological Observations of the Erythrocyte Sedimentation Rate in Dogs Affected by Leishmaniosis and Monitored During Antileishmanial Treatment

**DOI:** 10.3390/ani15121716

**Published:** 2025-06-10

**Authors:** George Lubas, Saverio Paltrinieri, Roberto Amerigo Papini, Ilaria Lensi, Silvia Benali, Oscar Cortadellas, Alessandra Fondati, Xavier Roura, Eric Zini

**Affiliations:** 1 Clinica Veterinaria Colombo, VetPartners Italia, V.le Colombo 153, Lido di Camaiore, 55041 Lucca, Italy; lensi.ilaria@gmail.com; 2 Department of Veterinary Medicine and Animal Sciences, University of Milan, Via dell’Università 6, 26900 Lodi, Italy; saverio.paltrinieri@unimi.it; 3 Department of Veterinary Sciences, University of Pisa, Viale delle Piagge 2, 56124 Pisa, Italy; roberto.amerigo.papini@unipi.it; 4 MYLAV, Laboratorio La Vallonea, Via G. Sirtori, 9, Passirana di Rho, 20017 Milano, Italy; silviabenali@laboratoriolavallonea.it; 5 Hospital Clínico Veterinario, Universidad CEU Cardenal Herrera, 46115 Valencia, Spain; oscar@vetgermanias.com; 6 Veterinaria Cetego, Via M.C. Cetego 20, 00177 Roma, Italy; alessandrafondati@gmail.com; 7 Hospital Clínic Veterinari, Universitat Autònoma de Barcelona, 08193 Bellaterra, Spain; xavier.roura@uab.es; 8 AniCura Istituto Veterinario Novara, Strada Provinciale 9, 28060 Granozzo con Monticello, Italy; eric.zini@anicura.it; 9 Department of Animal Medicine, Production and Health, University of Padova, Viale dell’Università 16, 35020 Legnaro, Italy; 10 Clinic for Small Animal Internal Medicine, Vetsuisse Faculty, University of Zurich, Winterthurerstrasse 260, 8057 Zurich, Switzerland

**Keywords:** canine, erythrocyte sedimentation rate, leishmaniosis, inflammatory markers, immune response markers

## Abstract

The erythrocyte sedimentation rate (ESR) is used in canine medicine in several disorders, especially to evaluate the levels of inflammation. ESR is a valid inflammatory marker in canine leishmaniosis (CanL), being markedly increased in sick dogs. This study evaluated the ESR together with several other inflammatory and immune response markers in 43 dogs affected by severe leishmaniosis, treated with antileishmanial treatment. Dogs were monitored at the beginning (T1) of treatment, in the middle (T2) of treatment, and 7–10 days after the end of the treatment (T3). The antileishmanial treatment was based on meglumine antimoniate and allopurinol for four weeks plus adding prednisolone at the anti-inflammatory dosage progressively tapered within the T2 set point. The ESR was measured and compared to immune and/or inflammatory markers (C-reactive protein, fibrinogen, ferritin, gamma-globulins, IgG). ESR levels were statistically reduced during treatment and significantly decreased at the end of the treatment. This came along with an improvement in other blood markers. This study shows the utility of ESR as a point-of-care test that can be used to monitor the response of dogs to antileishmanial treatment.

## Simple Summary

The erythrocyte sedimentation rate (ESR) has been increasingly used in canine medicine to assess inflammation levels. In this study, ESR was assessed in 43 dogs affected by severe leishmaniosis and treated with an antileishmanial treatment protocol based on antimonial and allopurinol for four weeks. This was performed to evaluate this inflammatory marker’s response to treatment. All dogs were classified according to the clinical staging proposed by the Canine Leishmaniosis Working Group (CLWG) and were examined at the beginning (T1) of treatment, in the middle (T2) of treatment, and 7–10 days after the end of the treatment (T3). ESR was compared to several other inflammatory and immune response markers typically investigated in dogs with leishmaniosis. During the follow-up, ESR, C-reactive protein, fibrinogen, and ferritin (as inflammatory markers), as well as gamma-globulins and Immunoglobulin G (as immune response markers), decreased significantly in response to treatment. ESR values may therefore help to stage the severity of canine leishmaniosis and to monitor the response to antileishmanial treatment.

## 1. Introduction

The erythrocyte sedimentation rate (ESR) is a commonly used blood test in the field of human hematology. The test provides a general indication of inflammation, measuring how quickly red blood cells in whole blood treated with anticoagulant settle in a standardized tube within one hour. To conduct the test, blood is typically placed in a vertical tube according to the standard procedure of Westergren. The distance between the top position of blood in the test tube and the point where red blood cells settle in one hour is measured and recorded in millimeters [1].

In human medicine, elevated ESR is frequently associated with various inflammatory diseases such as autoimmune disorders, cancers, infections, and acute or chronic tissue injuries [2,3,4]. ESR also accelerates in association with elevated concentrations of plasmatic proteins like fibrinogen and alpha-2-macroglobulin or glycoproteins like IgM, which have agglomerant properties [3,4]. Therefore, these proteins can act as a bridge between two cross-linking erythrocytes because they have high affinity for glycoproteins on the RBC membrane. In addition, these proteins can also function as neutralizers, as their positive charges nullify the negative charges of acid sialic residues on the RBC surfaces, which would otherwise repel each other [5,6]. Additional factors such as sex, age, albumin, and hematocrit play a role in the rate of sedimentation and the aggregation of red blood cells in human patients [6,7]. Nowadays, ESR measurement can easily be performed automatically with specific devices using an appropriately modified Westergren method and conducted either in clinics or in clinical pathology laboratories [8,9].

In the field of veterinary medicine, the use of ESR as an inflammatory marker has been recently reviewed in a wide range of canine diseases, including those derived from bacterial or parasitological infectious agents, from renal, urinary tract and orthopedic disorders, and based on an extended list of miscellaneous disorders [10]. The clinical utility of the ESR test lies in its ability to detect high, low, normal, increasing, or decreasing levels of inflammation. Therefore, ESR, in addition to being a tool with diagnostic applications, can help to monitor the inflammation during the course of a disease and to assess the effectiveness of a therapeutic treatment. Indeed, a decrease or an increase in ESR levels after the start of a treatment indicates, respectively, that the therapy is effective or fails to mitigate the causes of inflammation [10,11].

Recently, the ESR has been suggested as an additional laboratory parameter for the clinical evaluation of canine leishmaniosis (CanL) as a single infection [12,13] or as a coinfection with *Dirofilaria immitis* [14]. Furthermore, the use of ESR in feline [15,16] and equine medicine [17,18] has also been proposed, either for diagnostic or monitoring aims to assess the level of inflammation.

Previously, we reported that ESR values were significantly higher in a group of dogs infected with leishmaniosis and in groups of dogs with other inflammatory diseases compared to dogs infected with *Leishmania* but without clinical signs [13]. As an extension of the previous study and for the further investigation of the ESR as a helpful diagnostic tool in CanL, our goal was to measure ESR while monitoring antileishmanial treatment. For this purpose, we evaluated and compared the values of ESR and other immune and inflammatory markers in dogs affected by severe or very severe CanL at the beginning (T1), in the middle (T2), and after the end (T3) of the appropriate recommended antileishmanial treatment, following the clinical staging proposed by the Canine Leishmaniosis Working Group (CLWG) [19,20,21]. To our knowledge, this study is the first to evaluate the relationship between changes in ESR values and the leishmanicidal treatment protocol in dogs diagnosed with symptomatic CanL.

## 2. Materials and Methods

### 2.1. Study Design

A monocentric observational prospective study was performed between October 2021 and September 2024 in a private veterinary clinic. Given that blood and other biological samples were collected for routine diagnostic or monitoring purposes and the owners had signed a consent form that authorized the use of their data and the excess specimens for research purposes, formal approval from the University’s Ethical Committee was not required.

The study design was based on initial sampling, performed immediately before the first administration of antileishmanial treatment (T1), and on final sampling, performed 7–10 days after the last administration of antileishmanial treatment (T3) (43 samples were collected). Some dogs were also checked 14–18 days (T2) after the beginning of antileishmanial treatment (22 samples were collected).

Dogs included in the study were treated with subcutaneous injections of meglumine antimoniate at an initial dosage of 75–80 mg/kg for 4–5 days, then with 95–100 mg/kg divided in q12h, for a total of four weeks of treatment. Allopurinol was also administered at 15–20 mg/kg PO divided in q12h and reduced to 10–15 mg/kg PO divided in q12h. Then, xanthine crystals were seen in urine sediment during the follow-up. All dogs also received prednisolone at the dosage of 0.5–0.8 mg/kg PO q24h and this drug was rapidly tapered in about 14–18 days. The protocol used was the clinical decision of the two veterinarians (GL and IL) involved in the direct management of the antileishmanial treatment for each dog. Furthermore, all dogs received ectoparasite repellent such as collars impregnated with Imidacloprid-Flumetrin (Seresto®, Elanco Italia, S.p.A., Sesto Fiorentino, Italy) or spot-on topical pipettes (Advantix®, Elanco Italia, S.p.A., Sesto Fiorentino, Italy), according to the manufacturer’s indications and owner preferences, to prevent reinfections during the ongoing study and to avoid the transmission of Leishmania to sandflies.

### 2.2. Enrollment of Dogs

CanL-positive patients were examined by two veterinarians (GL and IL) who performed a thorough historical analysis with a complete physical examination and collected blood for a complete diagnostic workup, as specified below. Blood collected was split using three different vials: 1 mL in K3-EDTA, 2.5 mL in 3.8% sodium-citrate, and 6–7 mL in plain tubes.

Following the recommendations of the CLWG, the diagnostic workup to confirm CanL diagnosis included serology through the ELISA test, as described below, and/or cytology or qPCR on lymph node or bone marrow aspirates (according to the clinical presentation) [20,21]. Based on physical exams, abdominal ultrasound or thoracic radiographs were performed, as were other serological tests (these included immunofluorescence for *Ehrlichia canis*, *Anaplasma phagocytophilum*, *A. platys*, *Rickettsia* spp., *Babesia* spp., *Bartonella* spp., and *Hepatozoon* spp.)

Dogs were included in the study if clinical and clinico-pathological signs related to leishmaniosis, corresponding to stages C or D of the CLWG classification system, were present [20,21]. Additional inclusion criteria were the lack of comorbidities at the time of diagnosis and during the follow-up.

Exclusion criteria included antileishmanial treatments with antimonial, miltefosine, or allopurinol, performed three months prior to the study’s inclusion visit, and glucocorticoid treatment, performed a month prior to the study’s inclusion visit.

### 2.3. Laboratory Assays

All blood samples were collected from the jugular vein and promptly subdivided into three types of tubes specific to the following tests.

ESR was determined via 1 mL K3-EDTA vials (APTACA S.p.A., Canelli, Italy) using MINI-PET (DIESSE, Diagnostica Senese S.p.A., Siena, Italy). MINI-PET works without blood consumption. Thus, if an ‘error’ occurred while using this device (less than 1% of readings), the reading was repeated once again immediately after gently mixing the vial via inversion at least 10 times. The ESR samples were assayed within one hour of the blood collection and after the blood cell count had been carried out. The value of 10 mm/h was used as the upper limit of the reference interval in this study, as recommended by Militello et al. and according to the paper of Paltrinieri et al. [11,22]. Indeed, this value seems to be more appropriate for distinguishing between healthy and unhealthy dogs.

Routine hematology was performed on blood collected in EDTA tubes with an Idexx ProCyte^®^ Dx laser cell counter, (Idexx Laboratories, Westbrook, ME, USA). This was followed by the microscopical examination of May-Gründwald Giemsa-stained blood smears (MGG Quick Stain, Bio-Optika, Milan, Italy) by an experienced clinical pathologist [GL]. For this study, only hematocrit (Hct) and total leukocyte count (WBC) were recorded.

Plain tubes without any additive or gel were used to obtain serum by centrifugation of the samples for 10 min at 1500 G. Clinical chemistry was performed on this serum sample using an automated spectrophotometer (AU 5800, Beckman Coulter, Inc., Brea, CA, USA) with dedicated kit reagents. The following analytes were measured and recorded: C-reactive protein (CRP), iron, ferritin, haptoglobin (HPT), total proteins, albumin, immunoglobulin G (IgG), and immunoglobulin M (IgM). Serum protein electrophoresis (SPE) was performed on a capillary system (Capillarys Tera, Sebia, Evry Cedex, France) and the gamma-globulin (G-glob) percentage was recorded. Additionally, the following ratio was calculated and recorded—albumin/globulin (A/G)—dividing the value of albumin by total globulins.

Fibrinogen was measured by using an automated coagulometer (BCS XP, Siemens Healthcare Diagnostics, Marburg, Germany) on blood collected in 3.8% sodium citrate tubes and then by using dedicated kit reagents.

Serological assessmnt was carried out for leishmaniosis with the Leiscan^®^ *Leishmania* ELISA test (Hipra, Ecuphar Italia S.R.L., Milan, Italy), as previously reported by other authors [23,24,25]. The assay was carried out in serum samples following the manufacturer’s technical procedures. According to the package insert, the results were calculated and classified as follows: Razon (Rz) of the sample = sample Optical Density/control low positive sample Optical Density). The interpretation of Rz results were as follows: <0.7, negative; 0.7–1.5, suspected; 1.5–3, low positivity; 3–6, medium positivity; >6, high positivity. The test showed a specificity of 100% with a sensitivity of 92.5–98% in comparison studies [23,24] and was successfully used for a previous serological survey in dogs in Spain [25].

The real-time PCR (qPCR) was performed to detect and quantify *L. infantum* kinetoplast minicircle DNA using the primers and the protocol described by Castelli et al. 2021 [26]. The limit of detection was fixed at 100 copies of kinetoplast DNA.

### 2.4. Statistical Analysis

All the blood results collected over the study were assayed for normal distribution with the D’Agostino–Pearson test. All the values determined for each analyte were considered non-parametric data (because the caseload considered in this study was low) and were reported as median values and I and III interquartile ranges.

The Friedman test for paired samples was used to compare blood parameter values for samples collected at T1 and T3.

The Friedman test for related samples was used to compare blood parameters values for samples collected at T1, T2, and T3. If the pairwise comparison was statistically significant, a post hoc test was applied to the related variables (Wilcoxon test).

For each blood parameter, the percentage of values outside the reference interval (RI) was also calculated at all three checkpoints and the different percentages were compared using the Chi-square test.

Statistical analysis was performed using an Excel spreadsheet and a specific software (Analyse-it v. 6.15.4, Analyse-it Software Ltd., Leeds, UK). For all statistical tests, significance was set at *p* < 0.05.

## 3. Results

### 3.1. Number of Dogs, Samples Collected and Type of Treatment

After completion of initial clinical and clinicopathological data collection, a group of 43 dogs was followed for the entire cycle of leishmanicidal treatment. Leishmaniosis was diagnosed in three dogs by ELISA serology with high antibody titers, in one dog by bone marrow cytology, and in two dogs with qPCR using the lymph node or bone marrow, respectively; for the remaining 37 dogs, the diagnosis of CanL was made by a combination of ELISA serology with medium–high-antibody titers associated with the qPCR of lymph nodes (n = 8) or bone marrow (n = 9), or associated with the cytology of lymph nodes (n = 12) or bone marrow (n = 8). In one dog of the latter group, affected by nodular dermatitis, the diagnosis of leishmaniosis was also confirmed by skin cytology. All 43 dogs were examined at the time of the study’s enrollment visit, immediately before the first administration of antileishmanial treatment (T1), and 7–10 days after the end of antileishmanial treatment (T3). Twenty-two of these dogs were also examined midway through treatment (i.e., approximately 14–18 days after the start of antileishmanial treatment) (T2). Even if the clinical condition of each dog followed during the period of antileishmanial treatment was not scored as it was not included in the aim of this study, all dogs showed an improvement in clinical signs at the end of the cycle of treatment.

### 3.2. Number, Signalment, and Clinical Classification of Enrolled Dogs

Table 1 reports the signalment data for all the 43 enrolled dogs. Table 2 reports the main clinical features of the 43 CanL-positive patients along with the corresponding CLWG stages at the time of enrollment.

### 3.3. Comparison of Measurements at Times T1 and T3

Table 3 shows the values of the laboratory parameters examined in the group of 43 dogs affected by leishmaniosis, subjected to a complete course of antileishmanial treatment, and monitored at T1 and T3 during treatment.

Statistical analysis revealed that Hct, iron, albumin, and A/G were significantly higher at T3 than at T1, while ESR, CRP, fibrinogen, ferritin, total proteins, G-glob, and IgG were significantly lower at T3 than at T1. Also, HPT and IgM were significantly lower at T3 than at T1, though at a lower significance level. The only parameter that was almost unchanged and not significantly different was WBC.

### 3.4. Comparison of Measurements at T1, T2, and T3

Table 4 shows the values of the laboratory parameters examined in the group of 22 dogs affected by leishmaniosis and observed at times T1, T2, and T3 during the antileishmanial treatment.

Statistical analysis using Friedman test revealed that A/G, Hct, albumin, and iron were significantly higher at T3 than at T2 and T1, while ferritin, total proteins, G-Glob, IgG, ESR, CRP, and fibrinogen were significantly lower at T3 than at T2 and T1 (the listed parameters are reported in decreasing order based on the values corresponding to their statistical comparisons). WBC and IgM were the only parameters that were almost unchanged and not significantly different. In fact, it was also possible to notice the particular behavior of the HPT, which was higher at T2 than at T3.

The post hoc assay using the Wilcoxon test in paired checkpoints showed significant values for the following comparisons: in T1 vs. T2, there was a significant increase in Albumin, Hct, and A/G and a significant decrease in G-Glob, IgG, and ferritin; in T1 vs. T3, there was a significant increase in A/G, Hct, albumin, fibrinogen, and iron and a significant decrease in ferritin, total proteins, G-Glob, IgG, ESR, and CRP; in T2 vs. T3, there was a significant increase in A/G and Hct and a significant decrease in total proteins, G-glob, HPT, ESR, ferritin, and IgG.

### 3.5. Comparison of Percentages of Values Outside the Reference Interval

**Figure 1:**
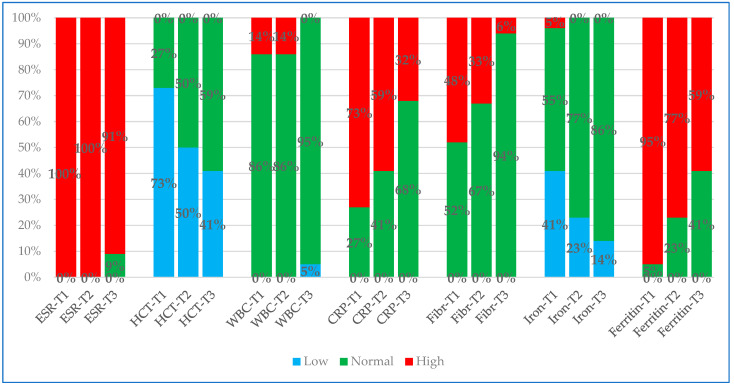
Partial list of analytes (ESR, Hct, WBC, CRP, fibrinogen, iron, and ferritin) investigated at T1, T2, and T3 time points, plotted and evidenced with different colors for percentage values lower than RI (blue), higher than RI (red) and within RI (green). Legend: ESR, erythrocyte sedimentation rate; Hct, hematocrit; WBC, total leukocyte count; CRP, C-reactive protein; Fibr, fibrinogen.

**Figure 2:**
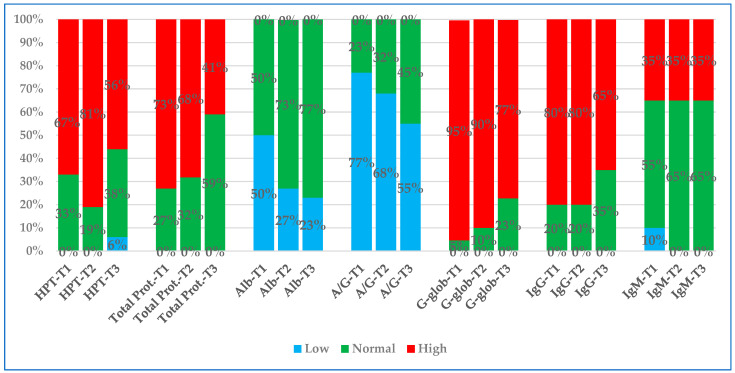
Partial list of analytes (HPT, total proteins, albumin, A/G, G-glob, IgG, and IgM) investigated at T1, T2, and T3, plotted, and evidenced with different colors for percentage values lower than RI (blue), higher than RI (red), and within RI (green). Legend: HPT, haptoglobin; Total Prot, total proteins; Alb, albumin; A/G, albumin–globulin ratio; G-glob, gamma-globulins in serum protein electrophoresis; IgG, immunoglobulin G; IgM, immunoglobulin M.

Figure 1 and Figure 2 graphically represent two partial lists of the various parameters examined at three time-points, showing whether they remained within the RI or whether they fell outside (above or below) the RI.

At T1, the percentage of values outside the RI for all parameters investigated was greater than 50% for ESR, ferritin, G-glob, IgG, A/G, Hct, CRP, total proteins, HPT, and albumin and less than 50% for fibrinogen, iron, IgM, and WBC (listed from the highest to the lowest percentage).

At T2, the percentage of values outside the RI was greater than 50% for ESR, G-glob, HPT, IgG, ferritin, total proteins, A/G, CRP, and Hct and less than 50% for IgM, fibrinogen, albumin, iron, and WBC (listed from the highest to the lowest percentage).

Finally, at T3, the percentage of values outside the RI was greater than 50% for ESR, G-glob, IgG, HPT, ferritin, and A/G and less than 50% for Hct, total proteins, IgM, CRP, albumin, iron, fibrinogen, and WBC (listed from the highest to the lowest percentage).

The Chi-square test performed for all three checkpoints, considering the percentage of values within or outside the RI, was only statistically significant for CRP (0.02) and ferritin (0.016).

## 4. Discussion

In this study, the ESR test was successfully used in dogs affected by severe or very severe leishmaniosis to monitor these patients during antileishmanial treatment. To the author’s knowledge, this is the first study to evaluate the relationship between ESR and the response to treatment in sick dogs diagnosed with leishmaniosis.

To assess the potential utility of the ESR in monitoring dogs affected by clinical and clinicopathological signs related to leishmaniosis during the antileishmanial treatment, a set of analytes related to protein dyscrasia and immune status, widely used to evaluate the severity of CanL, were evaluated simultaneously [27,28,29,30,31,32,33,34,35]. In addition, two parameters from the complete blood count were included. These were Hct, used to supply information about the RBC mass, and WBC, used to evaluate the total leukocyte count.

A comparison of the results recorded over time in dogs during this study revealed the progressive normalization of analytes, related to inflammation and anemia, that were impaired before treatment. Of particularly interest was ferritin, which is considered a prognostic marker in CanL [30,32].

Except for the WBCs and IgM, which did not change as expected in a chronic disease such as CanL, several differences were found in the comparison of the samplings from different times. Most of these differences are consistent with the normalization of the inflammatory/immune responses, although potentially influenced by the administration of anti-inflammatory dosages of prednisolone [36,37]. Some of these changes (for example G-glob) normalized at the middle of treatment (T2), while all the other analytes, including ESR, normalized from the middle to the end of treatment. This information could be useful to clinicians seeking to assess the improvement and the efficacy during the antileishmanial treatment.

In addition, although the extent of severity of clinical findings was not scored, according to the subtyping proposed by Cavalera et al. [12] and adopted by Da Silva et al. [38], all dogs showed a progressive improvement in clinical signs over time. ESR tended to follow the same trend, with a significant decrease at mid-treatment and a further decrease at the last observation, supporting the direct association between clinically active CanL and increased ESR as well as the subsequent role of ESR in predicting a good response to treatment when values tend to decrease over time after the initiation of the treatment.

As reported above, all dogs enrolled received, in addition to their specific antileishmanial treatment, a short course (14–18 days, shortly tapered) of oral prednisolone at a dosage generally considered anti-inflammatory at medium (0.2–0.5 mg/kg) or high (0.5–1.0 mg/kg) doses [39,40]. The use of glucocorticoids in the treatment of dogs with leishmaniosis is quite controversial, and few articles have been published on this topic. Some authors state that glucocorticoids can improve clinical signs and outcomes [41,42,43], while others advise against their use due to possible adverse effects including a reduction in the immune response against the CanL [44,45,46,47,48].

The use of immunosuppressive drugs including glucocorticoids in *Leishmania* affected dogs has been recommended when kidney glomerular disease is evidenced. The aim is to reduce immunomediate renal inflammation due to immune complex deposition rather than to decrease the formation and circulation of these complexes [45,46,49]. However, robust evidence supporting the use of immunosuppressive drugs in renal disease secondary to leishmaniosis is lacking. Expert opinions suggest the use of prednisolone (or even prednisone) at anti-inflammatory dosage (0.7 mg/kg orally, once a day for 3–10 days) in these cases [44,45,48,49].

Another application of glucocorticoids in dogs affected by leishmaniosis is established when concurrent pustular dermatitis is diagnosed [50,51]. Either prednisone or methylprednisolone were used at anti-inflammatory dosages, sometimes in combination with azathioprine or even cyclosporine. The purpose of this adjunctive treatment was to control pruritus and skin inflammation, and the treatment lasted longer than the use of antileishmanial drugs by up to several months, with progressive reductions in the dosages [50,51].

Finally, in very severe ocular manifestations of leishmaniosis, glucocorticoids are frequently adopted as topical treatment either, with a choice 0.1% of dexamethasone or 1% prednisolone. In the case of anterior and/or posterior uveitis, either a short course or tapered oral prednisolone was used (1.0 mg/kg twice a day for 5 days, the 1.0 mg/kg/day for 5 days, and 1.0 mg/kg every other day for 5 days) [52].

In this study, the two clinicians involved decided to use oral prednisolone for all dogs to reduce the inflammatory process induced by leishmaniosis and to overcome the local reaction at the skin site of the antimonial injection or other reported adverse reactions [53,54,55,56,57,58]. The collection of more clinical data about the effects of this glucocorticoid treatment could be useful to understand the benefit of its anti-inflammatory action, but this was not the aim of the study. It is possible that short-term therapy with an anti-inflammatory dosage of prednisolone may have somehow influenced the levels of blood parameters examined when assessing inflammatory status in this study, including ESR, by comparing the values at T1 with those at T2.

In the dog, the effect of glucocorticoids on the HPT serum levels is well known, and so the values collected for this parameter could be greatly influenced. In this study, the concentration of HPT was increased at T2 and was not significantly different from T1, while it was reduced at T3 in comparison to T1. The behavior of HPT at T2 could be influenced using prednisolone in the first 14–18 days of antileishmanial treatment [59,60]. It is also well known that glucocorticoids could increase the serum levels of iron [61]. In the evaluation at point three, we did not observe any statistical difference in the comparison of T1 vs. T2 and T2 vs. T3, while a statistical difference was seen in the T1 vs. T3 comparison. So, it seems that the serum levels of iron were mainly affected by the clinical improvement due to the antileishmanial treatment. The CRP serum values, on the contrary, were not affected by the administration of different glucocorticoids [60].

This clinical study enrolled and selected dogs in the field and had some typical limitations related to research design.

Our aim was to evaluate the utility of ESR in monitoring dogs with leishmaniosis during their specific antileishmanial treatment, the standard duration of which was set at 4 weeks. The last time-points for blood sampling in the cases included in this study were set at 7–10 days after the end of antileishmanial treatment. However, more interesting data could be collected from these dogs by further extending the observation period after the end of treatment [62].

Although the caseload included dogs of different ages, sexes, and attitudes, the potential effect of these variables was negligible since the aim of the study was to assess the differences in ESR values before and after treatment rather than comparing the results in different groups potentially biased by differences in age, sex or other pathophysiological variables that have been shown to potentially affect the ESR. Indeed, in humans, individual differences are non-inflammatory conditions that may influence ESR in some way and therefore ESR values may also vary depending on specific patient characteristics [6,7,63,64,65]. In veterinary medicine, only a few studies investigated whether patient characteristics of dogs influenced ESR [10]. Results showed that the influence of aging can lead to an increase in ESR [66,67] while sex is associated with significant differences in ESR values [68].

Enrolled dogs with clinical signs of leishmaniosis belonged to two different disease severity stages (C or D), as shown in Table 2. However, because of the low number of dogs that could be enrolled, results from both dogs in stage C and D were grouped together. Furthermore, clinical changes were not evaluated using a comprehensive scoring system for clinical signs associated with leishmaniosis, as previously reported by other authors [38,69,70,71]. Therefore, we cannot rule out the wide clinicopathological variability interfering with ESR levels.

It is important to note that a mild to severe decrease in Hct was observed in all enrolled and treated dogs. As previously reported in human medicine, as well as in dogs, ESR values generally tended to increase in anemic dogs. In humans, some authors have proposed an assessment of ESR measurements, corrected with an adjustment for Hct [65,72,73,74]. Similar investigations are also needed in veterinary medicine to investigate the impact of anemia on ESR values [10].

## 5. Conclusions

Understanding the evolution of clinicopathological data and laboratory findings in CanL during the therapeutic approach helps veterinarians to evaluate the response. The results of this study suggest the adjuvant role of ESR in monitoring the outcome of antileishmanial treatment in dogs with leishmaniosis, as ESR measurements have been shown to be good markers for the follow-up of these dogs. Therefore, our results may be useful in the application of laboratory criteria to evaluate the success of treatment in CanL and ESR may be included in the current laboratory parameters for routine clinical monitoring of dogs requiring courses of meglumine antimoniate plus allopurinol. It is suggested that ESR may potentially be one of the most valuable laboratory markers in clinics. It has point-of-care applications in the evaluation of CanL at presentation and is used during follow-up in small-animal veterinary clinics.

In the future, ESR results—always interpreted together with other clinical and clinico-pathological data—can be evaluated as potential reactive markers to predict the risk of the clinical relapse of CanL disease, occurring due to drug-resistant *L. infantum* strains. The aim of this is to monitor the stage of an infected dog without clinical signs, as well as to evaluate the efficacy of treatment in clinical trials conducted using already approved antileishmanial drugs such as miltefosine. This can be used alone or in combination with allopurinol or for the development and assessment of new drug candidates or adjuvant treatment for CanL.

## Figures and Tables

**Table 1:** Signalment data for 43 enrolled dogs.

Breed	N	Age	Sex	N
Mixed	19	Median 4 years	Males intact	26
English setter	5			
French Bouledogue	4	Range 2–14 years	Males castrated	4
Boxer	3			
Chihuahua, Siberian husky, and Pug (two each)	6		Females intact	4
American Staffordshire, Corso, Espagneul Breton, Dobermann, Poodle, Yorkshire terrier (1 each)	6		Females spayed	9

N—number of dogs.

**Table 2:** Main clinical features and CLWG clinical stage of 43 dogs with leishmaniosis.

Main Clinical Problem/s or Sign/s	N	
Weight loss and skin disease	7	
Skin disease and lymphadenopathy	5	CLWG
Weight loss and epistaxis	4	Stage D
Polyarthritis and lymphadenopathy and weight loss and enteropathy (two each)	4	N = 23
Skin disease, weight loss, and weight loss and enteropathy (one each)	3	
Weight loss	9	CLWG
Weight loss and enteropathy	4	Stage C
Weight loss and skin disease	3	N = 20
Enteropathy, enteropathy and lymphadenopathy, polyarthritis, and skin disease (one each)	4	

N—number of dogs.

**Table 3:** ESR values and other laboratory parameters in blood samples of 43 dogs affected by leishmaniosis collected at two time points (T1 and T3) and the relative statistical comparative evaluation (Friedman test).

Parameter (Units)	Reference Interval	N	Before Treatment (T1) Median (I–III IQR)	End Treatment (T3) Median (I–III IQR)	*p* *
ESR (mm/h)	<10	43	45.0 (21.3–55.8)	15.0 (12.0–38.0)	**<0.0001**
Hematocrit (%)	37.3–61.7	43	35.2 (27.8–40.5)	40.9 (35.8–45.3)	**<0.0001**
WBC (×10³/µL)	5.05–16.76	43	8.70 (6.25–11.60)	8.32 (6.81–11.65)	ns
CRP (mg/L)	0–0.15	43	8.4 (1.8–23.4)	1.2 (0.1–7.2)	**<0.0001**
Fibrinogen (mg/dL)	104–342	34	353 (258–512)	242 (196–317)	**<0.0001**
Iron (µg/dL)	70–270	43	80 (59–100)	99 (84–127)	**0.001**
Ferritin (ng/mL)	95–287	43	569 (327–708)	327 (201–392)	**<0.0001**
HPT (mg/dL)	18–117	34	203 (108–302)	138 (76–211)	**0.016**
Total proteins (g/dL)	5.5–7.6	43	7.82 (7.07–9.22)	7.02 (6.71–7.83)	**<0.0001**
Albumin (g/dL)	2.4–3.8	43	2.37 (1.91–2.69)	2.65 (2.28–2.82)	**<0.0002**
A/G ratio	0.6–1.3	43	0.41 (0.29–0.63)	0.62 (0.46–0.71)	**<0.0001**
G-glob (%)	5–15	43	30.0 (19.5–42.6)	19.5 (13.7–31.6)	**<0.0001**
IgG (mg/dL)	307–787	40	1494 (789–2274)	849 (526–1366)	**<0.0001**
IgM (mg/dL)	64–176	40	194 (126–260)	172 (115–234)	**0.027**

Legend: ESR, erythrocyte sedimentation rate; WBC, total leukocyte count; CRP, C-reactive protein; A/G, albumin–globulin ratio; HPT, haptoglobin, G-glob, gamma-globulins in serum protein electrophoresis; IgG, immunoglobulin G; IgM, immunoglobulin M; N, number of samples tested; I-III IQR, I-III interquartile range; ns, not significant; * in *p* column, significant values are reported in bold if values are statistically lower at T3 vs. T1 and bold plus underlined if the values are statistically higher at T3 vs. T1.

**Table 4:** ESR values and other laboratory parameters in blood samples in 22 dogs affected by leishmaniosis collected at three time points (T1, T2, and T3) and relative statistical comparative evaluation (Friedman test followed by post hoc with Wilcoxon test).

Parameter (Units) §	N	Before Treat. T1 Median (I–III IQR)	Middle Treat. T2 Median (I–III IQR)	End Treat. T3 Median (I–III IQR)	*p*	*p* * T1 vs. T2	*p* * T1 vs. T3	*p* * T2 vs. T3
ESR (mm/h)	22	42.5 (20.7–55.5)	39.0 (17.8–47.2)	15.0 (11.0–36.2)	**0.001**	ns	**0.0004**	**0.003**
Hematocrit (%)	22	33.0 (24.7–37.9)	36.8 (31.8–39.6)	38.1 (34.0–42.5)	**0.0004**	**0.007**	**0.0002**	**0.012**
WBC (K/µL)	22	9.05 (6.99–13.74)	9.19 (7.45–12.50)	9.13 (6.98–12.12)	ns	--	--	--
CRP (mg/L)	22	4.3 (1.1–10.5)	1.7 (0.2–9.8)	1.1 (0.4–2.6)	**0.035**	ns	**0.0007**	ns
Fibrinogen (mg/dL)	15	265 (245–418)	318 (245–374)	215 (191–270)	**0.015**	ns	**0.006**	**0.011**
Iron (µg/dL)	22	78 (59–109)	95 (71–119)	107 (88–130)	**0.042**	ns	**0.042**	ns
Ferritin (ng/mL)	22	586 (471–715)	347 (287–628)	341 (200–415)	**<0.0001**	**0.009**	**<0.0001**	**0.005**
Haptoglobin (mg/dL)	15	251 (114–306)	282 (190–327)	179 (83–292)	**0.002**	ns	ns	**0.001**
Total Proteins (g/dL)	22	8.93 (7.39–10.53)	8.40 (7.11–9.63)	7.24 (6.23–8.45)	**<0.0001**	ns	**<0.0001**	**<0.0001**
Albumin (g/dL)	22	2.40 (1.96–2.64)	2.59 (2.38–2.83)	2.65 (2.41–2.78)	**0.004**	**0.002**	**0.0004**	ns
A/G	22	0.39 (0.26–0.58)	0.50 (0.33–0.60)	0.58 (0.46–0.79)	**<0.0001**	**0.012**	**<0.0001**	**<0.0001**
G-glob (%)	21	41.8 (25.8–50.9)	30.7 (19.4–44.6)	20.5 (16.4–35.6)	**<0.0001**	**0.0003**	**<0.0001**	**<0.0001**
IgG (mg/dL)	18	2136 (791–2790)	1563 (857–2161)	1128 (476–1843)	**<0.0001**	**0.003**	**<0.0001**	**0.008**
IgM (mg/dL)	18	131 (80–191)	145 (108–194)	151 (108–179)	ns	-	-	-

Legend: §, reference ranges are reported in Table 3 as there are the same; ESR, erythrocyte sedimentation rate; WBC, total leukocyte count; CRP, C-reactive protein; A/G, albumin–globulin ratio; G-glob, gamma-globulins in serum protein electrophoresis; IgG, immunoglobulin G; IgM, immunoglobulin M; treat., treatment; I-III IQR, I-III interquartile range; ns, not significant; -, not applicable; in *p* * column, the significant values are reported in bold if statistically lower and bold plus underlined if statistically higher for each comparison.

## Data Availability

Data about this research are available on request to G.L. and are covered by privacy.
